# Wing geometry of *Culex coronator* (Diptera: Culicidae) from South and Southeast Brazil

**DOI:** 10.1186/1756-3305-7-174

**Published:** 2014-04-09

**Authors:** Bruna Demari-Silva, Lincoln Suesdek, Maria Anice Mureb Sallum, Mauro Toledo Marrelli

**Affiliations:** 1 Faculdade de Saúde Pública, Universidade de São Paulo, Avenida Dr. Arnaldo, 715, São Paulo, Brazil, CEP 01246–904; 2 Instituto Butantan, Avenida Vital Brazil, São Paulo, 1500, Brazil, CEP 05509–300

**Keywords:** Coronator group, *Cx. coronator*, Wing geometric morphometrics, Cerrado, Atlantic forest

## Abstract

**Background:**

The Coronator Group encompasses *Culex coronator* Dyar & Knab, *Culex camposi* Dyar, *Culex covagarciai* Forattini, *Culex ousqua* Dyar, *Culex usquatissimus* Dyar, *Culex usquatus* Dyar and *Culex yojoae* Strickman. *Culex coronator* has the largest geographic distribution, occurring in North, Central and South America. Moreover, it is a potential vector-borne mosquito species because females have been found naturally infected with several arboviruses, i.e., Saint Louis Encephalitis Virus, Venezuelan Equine Encephalitis Virus and West Nile Virus. Considering the epidemiological importance of *Cx. coronator*, we investigated the wing shape diversity of *Cx. coronator* from South and Southeast Brazil, a method to preliminarily estimate population diversity.

**Methods:**

Field-collected immature stages of seven populations from a large geographical area in Brazil were maintained in the laboratory to obtain both females and males linked with pupal and/or larval exuviae. For each individual female, 18 landmarks of left wings were marked and digitalized. After Procrustes superimposition, discriminant analysis of shape was employed to quantify wing shape variation among populations. The isometric estimator centroid size was calculated to assess the overall wing size and allometry.

**Results:**

Wing shape was polymorphic among populations of *Cx. coronator*. However, dissimilarities among populations were higher than those observed within each population, suggesting populational differentiation in *Cx. coronator*. Morphological distances between populations were not correlated to geographical distances, indicating that other factors may act on wing shape and thus, determining microevolutionary patterns in *Cx. coronator*. Despite the population differentiation, intrapopulational wing shape variability was equivalent among all seven populations.

**Conclusion:**

The wing variability found in *Cx. coronator* populations brings to light a new biological problem to be investigated: the population genetics of *Cx. coronator*. Because of differences in the male genitalia, we also transferred *Cx. yojoae* to the Apicinus Subgroup.

## Background

*Culex* (*Culex*) *coronator* Dyar and Knab, 1906 belongs to the Coronator Group, which comprises six other species (*Culex usquatus* Dyar, 1918, *Culex usquatissimus* Dyar, 1922, *Culex ousqua* Dyar, 1918, *Culex camposi* Dyar, 1925, *Culex covagarciai* Forattini, 1965, and *Culex yojoae* Strickman, 1989) [1]. Accurate identification of these species depends on examining traits of the dissected male genitalia because both adult females and immature stages are morphologically indistinguishable. *Culex coronator* was described based on morphological characteristics of larva collected in St. Joseph District in Trinidad. Adult stages of this species were previously identified as *Culex secutor* Theobald. In subsequent years, several species were described and their similarities to *Cx. coronator* were observed [2-8]. Later, only *Cx. coronator*, *Cx. usquatus*, *Cx. usquatissimus*, *Cx. ousqua*, *Cx. camposi* and *Culex covagarciai* were considered valid species because of differences in male genitalia traits and geographical distribution. Morphological similarities of immature stages and adult females suggested that *Cx. coronator* comprises a species complex [9,10]. Recently, Harbach [1] proposed a preliminary classification for the genus *Culex* based on morphological characteristics and transferred *Cx.yojoae* to the Coronator Group.

*Culex coronator* has the largest distribution among species of the Coronator Group. It occurs in Central and South America (Argentina, Belize, Bolivia, Brazil, Colombia, Costa Rica, El Salvador, French Guiana, Guatemala, Honduras, Nicaragua, Panama, Paraguay, Peru, Suriname, Trinidad and Tobago, and Venezuela), Mexico and United States [11,12]. In the latter country, this species is considered an invasive species that had dispersed eastward [13-16]. *Culex coronator* occurs in a large range of larval habitats that vary from natural to artificial containers, temporary ground pools, in full and partial shade or full sun, in sylvatic, rural and urban environments [17]. In Brazil, it was found in anthropic environments [18]. Females are predominantly nocturnal, exophilic and low anthropophilic [19,20].

Females of this species have been found naturally infected with several arboviruses, including the Saint Louis Encephalitis Virus (SLEV) in Brazil and in Trinidad and Tobago, Venezuelan Equine Encephalitis Virus (VEEV) in Mexico [21] and West Nile Virus in the United States [22,23]. In southeast Brazil, few human cases of encephalitis caused by SLEV have been reported [24,25]. However, it is possible that the prevalence of SLEV infections is underestimated because most infections are either unnoticeable or misdiagnosed because of cross-reactivity among different flaviviruses, including Dengue Virus, which are endemic in Brazil [25]. Moreover, VEEV Subtype IF caused encephalitis in humans in Vale do Ribeira, southeastern Atlantic Forest, Brazil. Potential determinants of the epidemics in the region of Vale do Ribeira are fully unknown, including mosquito vector species [26].

Dujardin [27], believed that phenotypic variation can improve the understanding of epidemiological importance in medically important insects, once they were submitted to various control pressures, such as landscape changes. In this sense, geometric morphometrics can be used to quantify this variation. Currently, this method has been largely employed both in distinction of closely related species [28-31] as in microevolution studies [32-36]. Considering the lack of biological and ecological studies regarding *Cx. coronator*, and the epidemiological importance of the species, the main objective of this study was to address variability of wing morphology across a transect to better understand the population structure and microevolutionary patterns of *Cx. coronator* populations in South and Southeastern Brazil. The circulation of SLVE and VEEV in Brazil and the fact of Rio Grande do Sul State is a migration route of Northern Hemisphere birds, such research may be useful to advance the knowledge about this potential vector.

## Methods

### Mosquito collections

**Figure 1:**
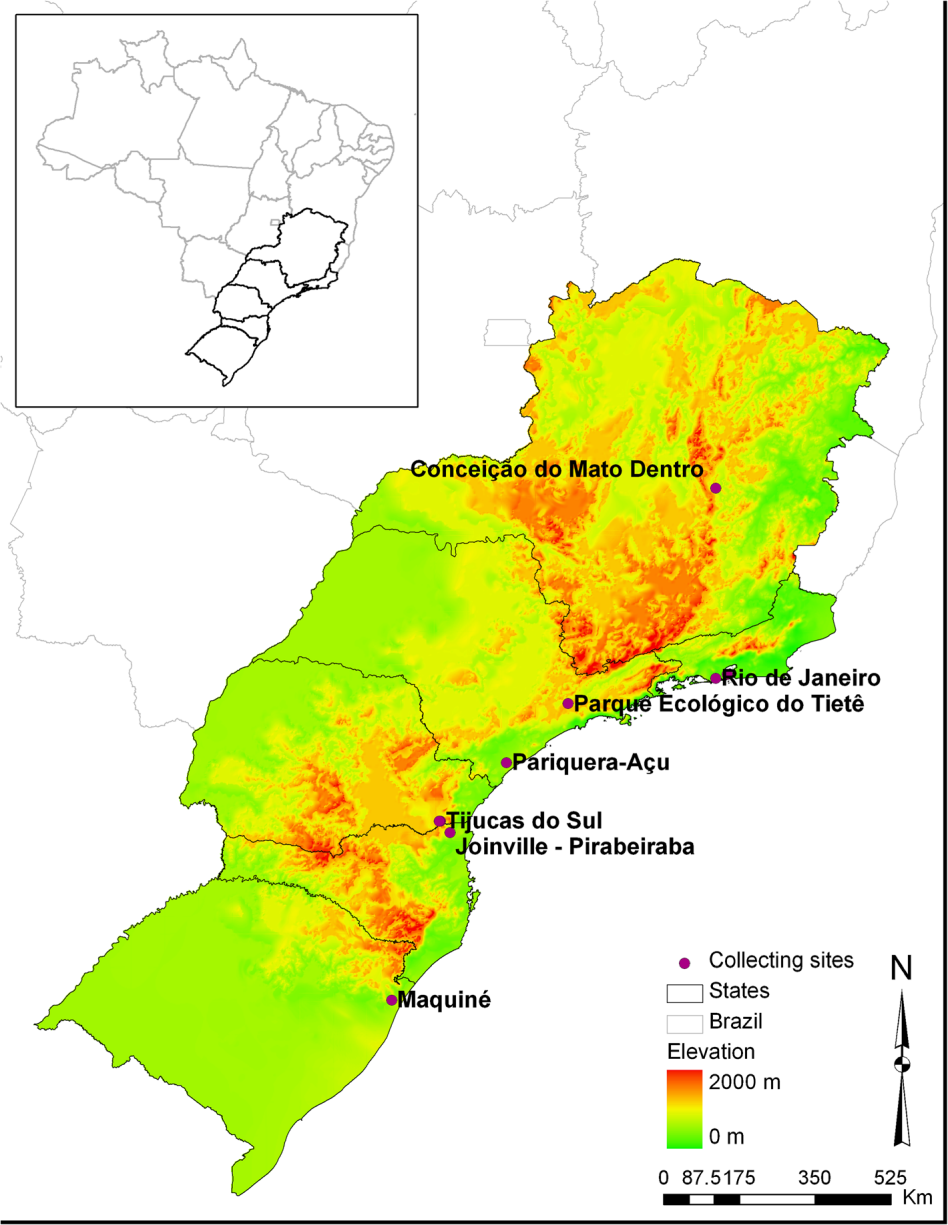
**Map of collecting sites (in dots) with indication of the elevation.** Lines indicate boundaries of the five States studied. Red zone between Minas Gerais, Rio de Janeiro and São Paulo States represents the location of Mantiqueira Mountains.

Mosquito immature stages were collected in 7 localities between 2010 and 2012 from October (spring) to January (summer) in localities in South and Southeastern Brazil (Table 1, Figure 1). Larvae and pupae were maintained in the laboratory at constant temperature and humidity to obtain males and females associated with their respective exuviae. Field collected larvae were transferred to double distilled water and artificially fed once a day with a mixture of finely ground Tetramin for tropical fish, Tetramin food for Beta fish, and dried pollen. A maximum of 30 larvae were kept in each receptacle with 400 ml of distilled water and fed with the same amount of food once a day. Water from each container was replaced daily to avoid development of pathogenic microorganisms. Male genitalia traits were employed for species identification and to support female identification.

**Table 1:** **Localities, specimens ID, States and geographic coordinates of sample collections of *Culex coronator *** **populations**

**Locality**	**State**	**Coordinates**
Pariquera-Açu	SP	24°44' S 47°49'W
Parque Ecológico do Tietê	SP	23°17’13.44”S 46°19’13.44”W
Rio de Janeiro	RJ	
Vargem Pequena neighborhood		22°35’11.40”S 43°16’29.89”W
Cosme Velho neighborhood		22°32’3.12”S 43°8’1.28”W
Conceição do Mato Dentro	MG	19°0’40.39”S 43°16’38.17”W
Tijucas do Sul	PR	25°56681’S 49°11326’W
Tijucas do Sul - Lagoinha	PR	25°34’0.52”S 49°6’47.74”W
Joinville - Pirabeiraba	SC	26°11065’S 48°59177’W
Maquiné	RS	29°40’26.58”S 50°12’50.22”’W

### Characterization of sampling localities

Pariquera-Açu municipality (PARI population) is situated in Ribeira Valley, Southeastern São Paulo State, within the Atlantic Forest domain. Because of intense anthropic pressures in the natural environment, most of the Atlantic Forest has either been removed or is under advanced secondary stage of succession. However, it remains better preserved in small forest fragments on the east coast of Brazil, with the largest continuous forest remaining in Southeastern São Paulo and northeast Paraná States. Larvae and pupae of *Cx. coronator* were collected in cattle ranches, ground pools, shallow ground depressions, ground holes, in natural and artificial habitats, in the proximities of forest.

Parque Ecológico do Tietê (PET population) is embedded in an urban environment, at the border of São Paulo and Guarulhos municipalities in the Tiete River floodplain. PET is a reforested area with 14 million squares meters of native floodplain vegetation. Pupae and larvae were collected in ground pools, in a restricted area in the park.

Rio de Janeiro municipality (RJ population) has a heterogeneous landscape in the Atlantic rainforest domains, with lowland coastal areas, mangrove and coastal mountains. Embedded in the southern border of the city, in the coastal mountains, there is a secondary forest preserved area named Tijuca Forest that is part of the Serra do Mar biodiversity corridor. *Culex coronator* specimens were captured in natural and artificial ground pools in two separated localities. One locality was a rural area situated in the proximity of the foot of the massif of Pedra Branca, which is also part of Serra do Mar biodiversity corridor. The second collection was carried out in an urban area, in Serra da Carioca, Tijuca Forest.

Conceição do Mato Dentro municipality (MG population) is located in the eastern portion of the Serra do Cipó and Espinhaço Mountains. It is inside a large ecological corridor formed by a mountain range that runs from the central region of Minas Gerais State, to the north of Bahia State. This is a transition area between Interior Atlantic Forest and Cerrado, with pastures as the major environmental modifier. Immatures of *Cx. coronator* were found in animal tracks and in rock holes near waterfalls.

Tijucas do Sul and Joinville municipalities (PR and SC populations) are embedded in a transition region between dense and semidecidous rain forests, with the family agriculture as the major environmental modifier. Sampling in Paraná State was in the first plateau [37] along the sides of secondary, dirty roads, in altitudes above 900 m. In contrast, in Santa Catarina States, immature collections were conducted in lower altitude areas (207 meter altitude) along the Atlantic coast.

Maquiné municipality (RS population) is a northeast rural area in Rio Grande do Sul State, in the south limit of the Atlantic Forest domain. Maquiné includes forest preserved areas, for instance, the Biological Reserve of Serra Geral and Rota do Sol, and it is situated in a transition zone between the coastal lowlands and the slopes of Serra Geral mountain. The vegetation in the area is characterized by a dense rain forest in the east and semidecidous forest in the west side Serra Geral. *Culex coronator* larvae and pupae were collected in rural areas, in marshes and in artificial man made ground holes.

### Wing preparation and data collection

**Figure 2:**
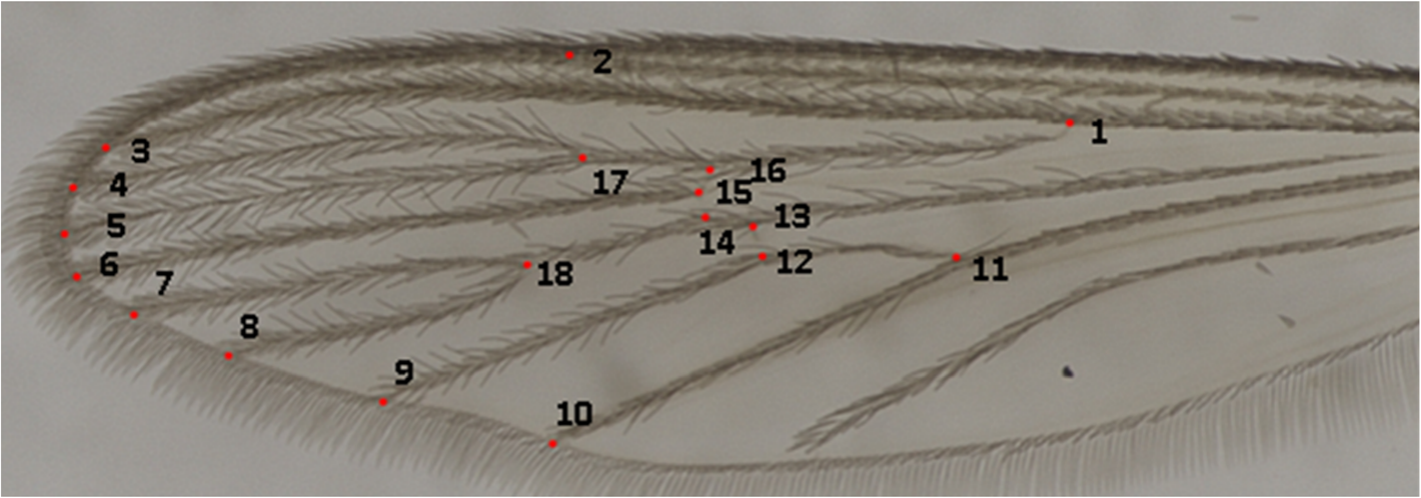
**Wing of *Cx. coronator *** **showing the 18 landmarks used in the morphometrics analysis.**

Left wings of the adult females were removed and mounted on flat microscope slides under a 0.08 - 0.12 mm glass coverslip using Canada balsam. Microscope slides of wings, male genitalia, pupal and fourth-instar larval exuviae were deposited in the Coleção Entomológica de Referência, Faculdade de Saúde Pública, Universidade de São Paulo (FSP-USP), Brazil. Wing images were obtained with a Leica M205C, under 50x magnification. In each image, 18 landmarks (Figure 2) were digitalized using tpsDig software v2.0 [38]. *Culex coronator* populations included in the study were obtained in Pariquera-Açu (PARI, n = 23) and Parque Ecológico do Tietê (PET, n = 27) - both in São Paulo State- and in the States of Rio de Janeiro (RJ, n = 23), Minas Gerais (MG, n = 27), Santa Catarina (SC, n = 29), Paraná (PR, n = 24) and Rio Grande do Sul (RS, n = 23). Additionally, 23 samples of *Culex quinquefasciatus*, from Santa Vitória do Palmar municipality [39], Rio Grande do Sul State, were employed as outgroup in the Neighbour-Joining analyses .

### Morphometric approach

To investigate the distribution of wing shape and to address specific questions about intraspecific heterogeneities in *Cx. coronator*, a generalized Procrustes distance analysis was performed in COV [40]. A Procrustes distance matrix was constructed and employed to generate a neighbor-joining (NJ) topology in PHYLIP version 3.7 [41]. NJ topology was visualized in TreeView [42]. Correlation between Procrustes and geographic distance was addressed using the Mantel’s Test.

The overall size of left wing was estimated by the centroid size (CS) in MOG [40]. CS was estimated according to Bookstein [43]. Descriptive statistic graphs were constructed in Statistica 7 (StatSoft). The ANOVA and Tukey-Kramer tests were employed to ascertain the significance of differences between averages of the CS among populations. Both tests were conducted in GraphPad InStat v.3 (GraphPad Software, San Diego California USA). To determine potential association between mean centroid size and latitudinal variation, a simple regression analysis was conducted in Statistica 7 (StatSoft). The allometric effect was verified and removed from further analyses employing the regression of Procrustes coordinates (dependent variable) against CS (independent variables).

To address variation in wing shape among groups, a discriminant analysis of principal components derived from Procrustes superimposition method was performed using Statistica 7 software. Only the set of 12 first Relative Warps were employed in discriminant analysis, since they explained 94.25% of the total variation of wing shape. Accuracy of population recognition yielded by morphometrics was evaluated by a reclassification test. For this test, the similarity of wing shape of each individual was compared with the average of wing shape of each population, using Procrustes distances in MorphoJ software [44]. In order to find the two most divergent wing shapes, we performed a Canonical Variate Analysis (CVA) also in MorphoJ program. The most distant samples in morphospace of the CVAs were taken and, then, an analysis of spline plots in tpsSplin v3.2 program was carried out, to assess wing deformations between those specimens. Morphological diversity was estimated by the “amount of dispersion” of the individuals in the morphospace of principal components (PCs). The dispersion was estimated using the following procedure. The Positional (Cartesian) coordinates of plots in the morphospace of PCs (each one corresponding to a single mosquito individual) were digitalized using TpsDig software, similar to the wing landmarks. The centroid size vector of a set of individuals in the morphospace (a population) was estimated in TpsRelW software [38]. Following this, the value of this vector was used to evaluate morphological diversity within each population (MD). The amount of dispersion of individuals (of a single set) in the morphospace of PCs is proportional to the morphological variability of that population set.

## Results

### Wing size

**Figure 3:**
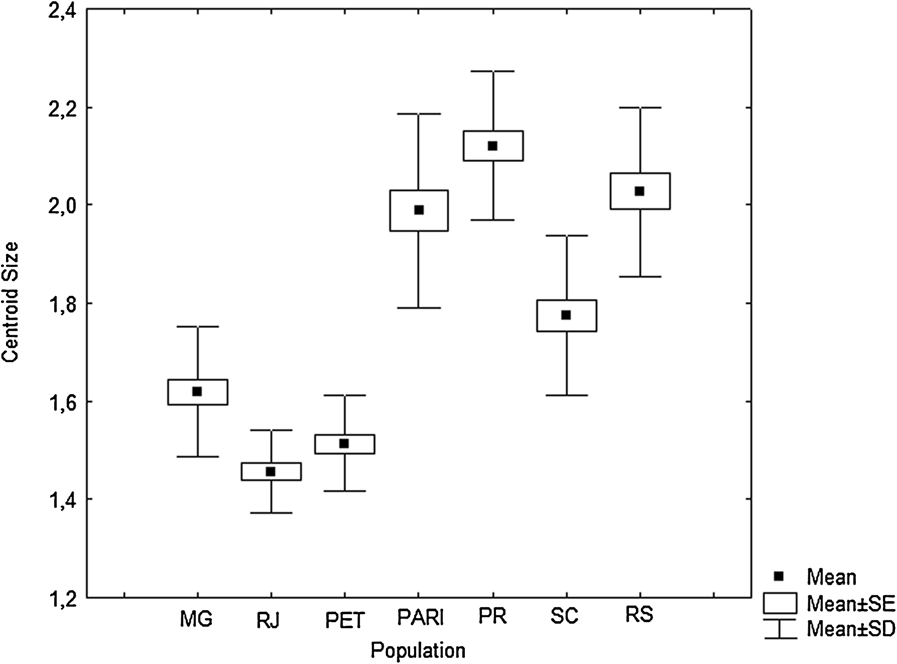
**Descriptive statistics of centroid sizes of the seven populations of *Culex coronator.***

**Figure 4:**
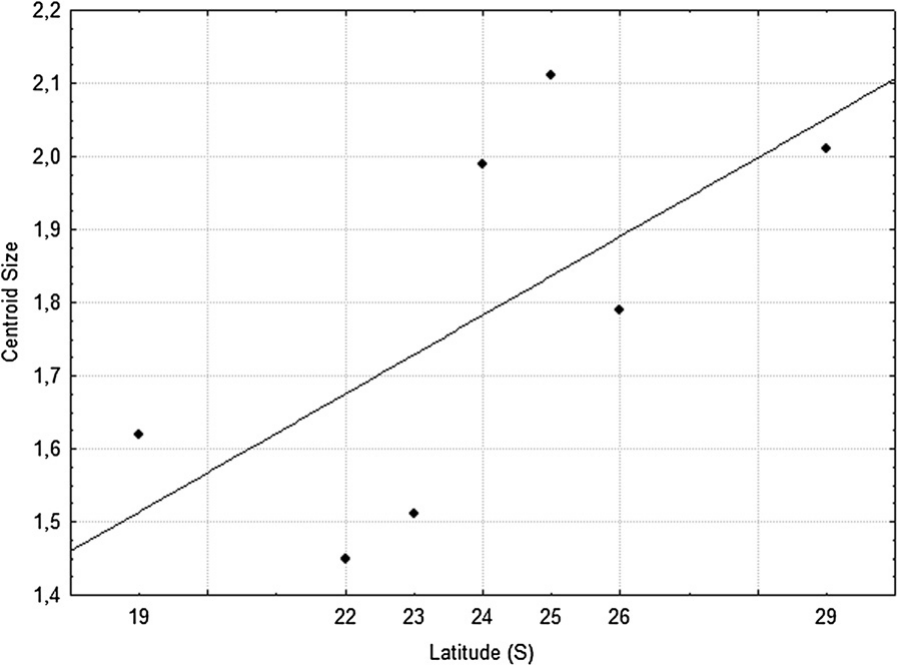
**Simple linear regression between mean centroid sizes and latitudes.** Mean CS are in mm and latitudes are in degrees.

CS values ranged from 1.36 mm to 2.25 mm in *Cx. coronator* populations employed in this study. The lowest mean for the CS was found in RJ (1.45 mm) populations, while the highest mean CS was observed in PR (2.11 mm). The remaining populations had the following CVs means: 1.62 mm (MG), 1.99 mm (PARI), 1.51 mm (PET), 1.79 mm (SC) and 2.01 mm (RS). The population with the highest variation was PARI (1.79 mm to 2.2 mm) (Figure 3). Statistical significance of the differences between mean centroid sizes among populations is in Table 2. Results of the simple regression analysis showed a positive association between latitudinal gradient and mean centroid size (Figure 4), with moderate correlation (R^2^ = 0.50) that was not significant (P = 0.072).

**Table 2:** ANOVA test for the significance of mean Centroid Size differences among populations

	**MG**	**PARI**	**PET**	**RJ**	**RS**	**SC**	**PR**
MG							
PARI	**<0.001**						
PET	>0.05	**<0.001**					
RJ	**<0.01**	**<0.001**	>0.05				
RS	**<0.001**	> 0.05	**<0.001**	**<0.001**			
SC	**<0.001**	**<0.001**	**<0.001**	**<0.001**	**<0.001**		
PR	**<0.001**	** < 0.05**	**<0.001**	**<0.001**	>0.05	**<0.001**	

For P < 0.05 the differences were considered significant (in bold).

### Wing shape

**Figure 5:**
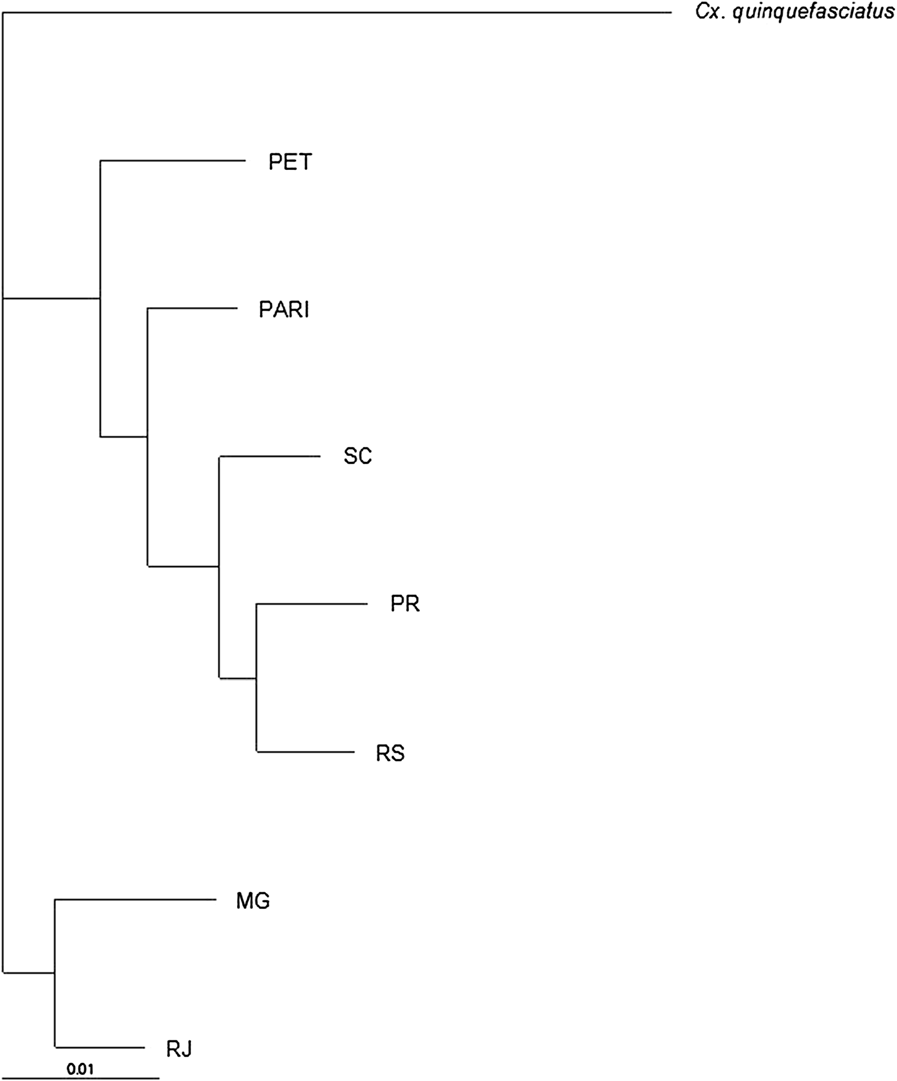
**Neighbor-joining phenogram generated from a Procrustes distance matrix of the seven populations of *Culex coronator *** **from Southeastern and Southern, Brazil.**

The NJ tree generated using the Procrustes distance matrix (Figure 5) showed two population clusters. One cluster included populations from MG and RJ, whereas the second cluster was comprised of individuals from the remaining 5 populations.

**Figure 6:**
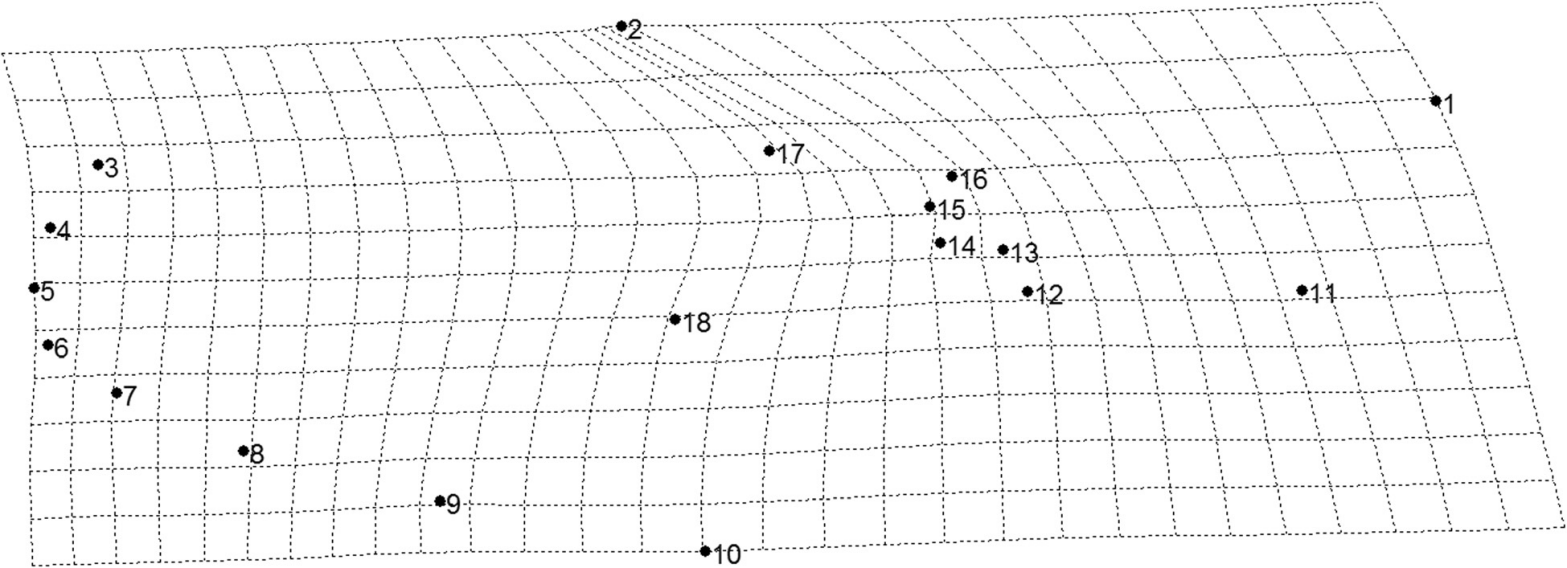
Thin plate splines showing deformations between the most divergent wings in CVA morphospace (from SC and RJ populations) among all populations analyzed.

Likewise, southern populations (PR, SC and RS) showed the highest similarity, with PR and RS morphologically more similar to each other than to SC. PARI population was found closer to southern than to southeastern populations, whereas PET was the most distinct grouping within the cluster. The most divergent samples from CVA were from RJ and SC. Thin Plate Splines analysis showed most deformations in the medial part of the wing (landmarks 13, 14, 15 and 16) and landmark 2 - Figure 6. Results of regression analysis carried out to investigate evolutionary integration in the wing shape and wing size were statistically significant, with moderate allometric effect (5.11%). After removing the allometric effect, results of the permutation test showed that differences between MG and the remaining populations were all statistically significant (Table 3). Results of cross-validated reclassification tests are in Additional file 1. Intrapopulational diversity indexes for all 7 populations were similar (see Additional file 2).

**Table 3:** P-values from discriminant analysis among groups

	**MG**	**RJ**	**PET**	**PARI**	**PR**	**SC**	**RS**
RJ	**0.0036**						
PET	**<0.0001**	0.0534					
PARI	**0.0162**	0.6373	**0.0171**				
PR	**0.0005**	0.5578	0.1889	0.2959			
SC	**0.0005**	**0.0009**	0.1360	**0.0002**	**0.0069**		
RS	**<0.0001**	0.0599	0.9801	0.6373	0.1976	0.1591	

For P < 0.05 the differences were considered significant (in bold).

## Discussion

Wing morphology analysis of *Cx. coronator* populations collected from South to Southeastern Brazil were polymorphic showing population structured with microevolutionary patterns. Although Procrustes distances were not correlated with geographical distances, our results clearly separate *Cx. coronator* into two subgroups that are correlated to the macro-geographical regions where samples were collected. As such, one subgroup includes individuals from the “southeastern populations” (RJ and MG), whereas the second subgroup is composed of the “southern populations” (PARI, PET, PR, SC and RS). Morphological differentiation between both southeastern and southern *Cx. coronator* populations may be explained by the presence of high altitude areas of Serra da Mantiquera, along the borders of São Paulo, Rio de Janeiro and Minas Gerais States. The mountain range may preclude dispersal of *Cx. coronator* through those areas, causing isolation of RJ and MG from the southern populations. Thus, it is likely that a shallow, incipient speciation process is occurring in those two population subgroups.

Considering the subgroup composed of RJ and MG populations, we found that wing shape variability within MG is slightly higher than in the RJ population. It is noteworthy that representatives from MG populations were collected in the ecotone between two biodiversity hotspots, Cerrado and Atlantic Forest, within the ecological corridor of the Cerrado. In the ecotone, important ecological and evolutionary processes may occur and induce genetic diversity and wing shape variability, in response to highly heterogeneous physiognomies and landscapes present in the zone [45,46]. In contrast, the RJ population was from localities embedded in the coastal Atlantic Forest where ecological determinants are less heterogeneous than in the transition zone between Cerrado and Atlantic Forest. In a study that used geometric morphometric methods to investigate evolutionary association between biome and female wing shape in *Anopheles darlingi*, a primary mosquito vector of human *Plasmodium*, Motoki *et al.*[33] suggested that wing shape variations could be caused by distinct selective pressures present in Cerrado and interior and coastal Atlantic Forest. Based on both the results of the present study and the findings of Motoki *et al*. [33], it is possible that shallow wing shape heterogeneities between MG and RJ populations were generated by evolutionary pressures present in the Cerrado and Atlantic Forest, rather than isolation by distance. Similarly, a study with dragonfly associated heterogeneities observed in the forewing shape with selective pressures represented by distinct landscapes, with the wing becoming broader from forested to open areas [47]. Although the effect of selection on the wing landmarks is not fully understood, wing shape characteristics have been effectively employed to address population structure associated with landscapes, habitat split and isolation in several arthropod species [32-34,48,49].

In the NJ topology, within the clade composed of southern populations (Figure 5), PET was separate from the remaining southern population, which formed a secondary grouping (PARI, PR, SC, and RS). Accordingly, PARI population was demonstrated to be morphologically more similar to SC than to PET and PR populations. Environmental differences between PET and remaining southern sampling localities, in contrast, are enormous. For instance, samples from PET populations were obtained in a recently reforested area of semi deciduous rain forests in the floodplain of the highly polluted Tiete River, at the border of São Paulo and Guarulhos municipalities. This area suffered and is still under strong ecological pressures caused by intensive anthropic changes in the environment and pollution. Distinctly, individuals from PARI were captured in the proximity of the borders of São Paulo and Paraná States, in a rural area embedded within the coastal Atlantic Forest (Figure 1). On the other hand, ecological exposure factors are similar for PR, SC and RS populations. Field collections in Paraná, Santa Catarina and Rio Grande do Sul States, were in areas of semi-deciduous forest in a subtropical climate. Similar ecological conditions may facilitate both the establishment and the dispersion of individuals that are better adapted to environmental characteristics present in semi-deciduous forest. Consequently, gene flow is still occurring between those populations which are undergoing similar evolutionary pressures. However, ecological factors between PARI and SC are distinct and did not explain a close morphological similarity between them. Likely, the similarity may be caused by retention of ancestral wing shape polymorphism. In spite of being able to occupy a wide range of larval habitats in heterogeneous landscapes within distinct biomes physiognomies, wing shape diversity in *Cx. coronator* was similar both within and in southern populations. So, it is possible that dispersal and gene flow, in both cases, are being somehow facilitated.

Our results also showed a positive association between wing size and latitude, with the average centroid size increasing in higher latitudes. According to Bergman’s rule, endothermic vertebrates in higher latitudes tend to be bigger, as a result of colder climates. Later, Ray [50] stated that ectothermic invertebrates such as insects also would fit this rule. However, Mousseau [51] suggested that the body size in ectothermic animals decreased with increasing latitude, and consequently, with decreasing temperature, and proposed the reverse Bergmann’s rule. Recently, Blanckenhorn & Demont [52] demonstrated that, for arthropods, both theories occurs in nature. Meanwhile, larger insects, with longer development time, follow the reverse Bergmann’s rule, while smaller insects, with faster development periods, would fit the Bergman’s rule. Here, the analyzed sample populations of *Cx. coronator* clearly follows the Bergman’s rule.

Currently, association between wing and body size in vectorial capacity is not completely understood. Meanwhile, body size can have a major effect on the longevity, fecundity and size of blood meal of vector-borne disease insects [53,54], influencing the vectorial capacity of populations. Additionally, several studies have shown that heterogeneities in the wing size may be a plasticity response to environmental factors, such as relative humidity, temperature and food availability [55-57]. Nevertheless, morphological plasticity has an important role in ecology because populations that have higher adaptative responses can occupy larger ecological niches [58]. Consequently, a better understanding of the causes and consequences of phenotypic plasticity may be important to explore mechanisms of evolution. Moreover, plasticity is a result of evolutionary integrations between environmental factors and genotypes [59-61]. Thereby, environmental determinants can facilitate the perpetuation of some populations under directional selection [59].

Hitherto, regional dissimilarities did not indicate *Cx. coronator* as a group of cryptic species. Nevertheless, we cannot discard the hypothesis that the polymorphisms observed among populations may be the substratum of an incipient speciation process. Recently, Harbach [1] compiled morphological data to propose a preliminary classification for the genus *Culex*, including the subgenus *Culex*. Accordingly, the Coronator Group is composed of *Cx. camposi*, *Cx. covagarciai*, *Cx. ousqua*, *Cx. usquatissimus*, *Cx. usquatus* and *Cx. yojoae*. The latter species was described by Strikman [62] based on female, male, fourth-instar larva and pupae from La Joya, Cortes Department, Honduras. Comparisons among characteristics of the male genitalia of *Cx. yojoae* and the remaining Neotropical *Culex* (*Culex*) species show that *Cx. yojoae* is more similar to *Culex chidesteri* and *Culex pseudostigmatosoma* than to any species of both the Coronator Group. It is noteworthy that *Cx. chidesteri* was included in the Apicinus Subgroup, whereas *Cx. pseudostigmatosoma* was considered a species of the Tarsalis Subgroup, both subgroups of the Pipiens Group, by Harbach [1]. Presence of foliform *g* setae in the distal division of the subapical lobe of the gonocoxite, the shape of the ventral and dorsal divisions of the lateral plate of the phalosome and the presence of spatulate spines in the paraproct crown suggest that *Cx. chidesteri*, *Cx. yojoae* and *Cx. pseudostigmatosoma* may comprise a species complex. We are herein transferring both *Cx. yojoae* and *Cx. pseudostigmatosoma* to the Apicinus Subgroup. Notwithstanding, it is obvious that the subgenus *Culex* needs further and more detailed studies before proposing a new internal classification for it.

## Conclusions

Results of the present study show that *Cx. coronator* is a polymorphic species with morphological dissimilarities in both wing shape and size. Considering that wing shape has a polygenic basis [63], differences among populations may be caused by polymorphisms in those genes. Moreover, it is plausible to suppose that heterogeneities in wing size are correlated to plasticity response to ecological determinants of the larval habitats. In both cases, further studies, including molecular data, will be necessary to provide a more comprehensive understanding of the variability of the wing traits of a largely dispersed mosquito vector species. While identifying the C*ulex* specimens collected for the present study, we checked our samples with all species of the Coronator Group as defined by Harbach [1]. In considering only adult females characteristics discussed by Strikman [62], he concluded that *Cx. yojoae* was a species of the Coronator group. However, most morphological traits used to identify species of the genus *Culex* are from male genitalia, thus we concluded that there is a misinterpretation in Harbach’s [1] revision of C*ulex.* Consequently, we strongly suggest that *Cx. yojoae* belongs to Apicinus Subgroup due to the similarities between the male genitalia of this species and *Cx. chidesteri.*

## Competing interests

We declare that we don’t have competing interests.

## Authors’ contributions

MAMS, BDS, MTM conceived the study. BDS collected material in field and morphometric data. BDS and LS performed wing morphometric analyses and interpretation of the results. All authors read and agreed with the final version of the manuscript.

## Supplementary Material

Additional file 1 **Cross-validated reclassification of individuals from the seven populations clusters of *Culex coronator.*** Values indicate correct classification and are represented both in absolute values and in percentage. Click here for file

Additional file 2 Morphological intradiversity of the seven populations studied. Click here for file
