# Supplementary material for: Wing geometry of Culex coronator (Diptera: Culicidae) from South and Southeast Brazil
Source: Parasit Vectors. 2014 Apr 9;7:174. doi: 10.1186/1756-3305-7-174 (PMC4113194; doi:10.1186/1756-3305-7-174)
Supplement: Additional file 1 — Cross-validated reclassification of individuals from the seven populations clusters of Culex coronator. Values indicate correct classification and are represented both in absolute values and in percentage. [file 1756-3305-7-174-S1.doc]

| Clusters | MG | PARI | PET | RJ | PR | RS | SC | Total |
| --- | --- | --- | --- | --- | --- | --- | --- | --- |
| MG | 19 (70.3%) | 8 (29.7%) |  |  |  |  |  | 27 |
| PARI | 7 (30.5%) | 16 (69.5%) |  |  |  |  |  | 23 |
| MG | 20 (74%) |  | 7 (26%) |  |  |  |  | 27 |
| PET | 6 (22.3%) |  | 21 (77.7%) |  |  |  |  | 27 |
| MG | 13 (48%) |  |  |  | 14 (52%) |  |  | 27 |
| PR | 10 (43.5%) |  |  |  | 13 (56.5%) |  |  | 23 |
| MG | 19 (70.3%) |  |  | 8 (29.7%) |  |  |  | 27 |
| RJ | 8 (33.3%) |  |  | 16 (66.7%) |  |  |  | 24 |
| MG | 19 (70.3%) |  |  |  |  | 8 (29.7%) |  | 27 |
| RS | 2 (8.7%) |  |  |  |  | 21 (91.3%) |  | 23 |
| MG | 21 (77.8%) |  |  |  |  |  | 6 (22.2%) | 27 |
| SC | 8 (27.6%) |  |  |  |  |  | 21(72.4%) | 29 |
| PARI |  | 21 (91.3%) | 2 (8.7%) |  |  |  |  | 23 |
| PET |  | 3 (11.2%) | 24 (88.8%) |  |  |  |  | 27 |
| PARI |  | 17 (74%) |  |  | 6 (26%) |  |  | 23 |
| PR |  | 6 (26%) |  |  | 17 (74%) |  |  | 23 |
| PARI |  | 14 (61%) |  | 9 (39%) |  |  |  | 23 |
| RJ |  | 9 (37.5%) |  | 15 (62.5%) |  |  |  | 24 |
| PARI |  | 18 (78.2%) |  |  |  | 5 (21.8%) |  | 23 |
| RS |  | 6 (26%) |  |  |  | 17 (74%) |  | 23 |
| PARI |  | 19 (82.6%) |  |  |  |  | 4 (17.4%) | 23 |
| SC |  | 11 (38%) |  |  |  |  | 18 (62%) | 29 |
| PET |  |  | 15 (56%) |  | 12 (44%) |  |  | 27 |
| PR |  |  | 11 (48%) |  | 12 (52%) |  |  | 23 |
| PET |  |  | 23 (85%) | 4 (15%) |  |  |  | 27 |
| RJ |  |  | 5 (20%) | 19 (80%) |  |  |  | 24 |
| PET |  |  | 20 (74%) |  |  | 7 (26%) |  | 27 |
| RS |  |  | 5 (21.7%) |  |  | 18 (78.3%) |  | 23 |
| PET |  |  | 19 (70.4%) |  |  |  | 8 (29.6%) | 27 |
| SC |  |  | 10 (34.5%) |  |  |  | 19 (65.5%) | 29 |
| PR |  |  |  | 8 (34.8%) | 15 (65.2%) |  |  | 23 |
| RJ |  |  |  | 13 (54%) | 11 (46%) |  |  | 24 |
| PR |  |  |  |  | 14 (61%) | 9 (39%) |  | 23 |
| RS |  |  |  |  | 9 (39%) | 14 (61%) |  | 23 |
| PR |  |  |  |  | 17 (74%) |  | 6 (26%) | 23 |
| SC |  |  |  |  | 9 (31%) |  | 20 (69%) | 29 |
| RJ |  |  |  | 12 (50%) |  | 12 (50%) |  | 24 |
| RS |  |  |  | 8 (34.8%) |  | 15 (65.2%) |  | 23 |
| RJ |  |  |  | 15 (62.5%) |  |  | 9 (37.5%) | 24 |
| SC |  |  |  | 9 (31%) |  |  | 20 (69%) | 29 |
| RS |  |  |  |  |  | 19 (82.6%) | 4 (17.4%) | 23 |
| SC |  |  |  |  |  | 19 (65.5%) | 10 (34.5%) | 29 |

**Additional file 1.** Cross-validated reclassification of individuals from the seven populations clusters of *Culex coronator.* Values indicate correct classification and are represented both in absolute values and in percentage.
