# Supplementary figures and images for: Wing geometry of Culex coronator (Diptera: Culicidae) from South and Southeast Brazil
Source: Parasit Vectors. 2014 Apr 9;7:174. doi: 10.1186/1756-3305-7-174 (PMC4113194; doi:10.1186/1756-3305-7-174)

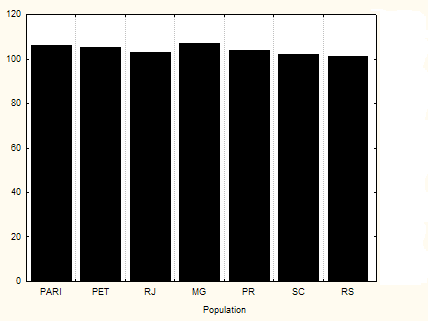

Supplement: Additional file 2 — Morphological intradiversity of the seven populations studied. [file 1756-3305-7-174-S2.png]
